# Comparative analysis examining patterns of genomic differentiation across multiple episodes of population divergence in birds

**DOI:** 10.1002/evl3.46

**Published:** 2018-03-15

**Authors:** Kira E. Delmore, Juan S. Lugo Ramos, Benjamin M. Van Doren, Max Lundberg, Staffan Bensch, Darren E. Irwin, Miriam Liedvogel

**Affiliations:** ^1^ Max Planck Institute for Evolutionary Biology Behavioural Genomics 24306 Plön Germany; ^2^ Edward Grey Institute, Department of Zoology University of Oxford OX1 3PS Oxford United Kingdom; ^3^ Lund University Department of Biology 223 62 Lund Sweden; ^4^ Biodiversity Research Center University of British Columbia V6T 1Z4 Vancouver British Columbia Canada

**Keywords:** Bird, genomic differentiation, genomic hitchhiking, islands of differentiation, population divergence, speciation

## Abstract

Heterogeneous patterns of genomic differentiation are commonly documented between closely related populations and there is considerable interest in identifying factors that contribute to their formation. These factors could include genomic features (e.g., areas of low recombination) that promote processes like linked selection (positive or purifying selection that affects linked neutral sites) at specific genomic regions. Examinations of repeatable patterns of differentiation across population pairs can provide insight into the role of these factors. Birds are well suited for this work, as genome structure is conserved across this group. Accordingly, we reestimated relative (*F_ST_*) and absolute (*d_XY_*) differentiation between eight sister pairs of birds that span a broad taxonomic range using a common pipeline. Across pairs, there were modest but significant correlations in window‐based estimates of differentiation (up to 3% of variation explained for *F_ST_* and 26% for *d_XY_*), supporting a role for processes at conserved genomic features in generating heterogeneous patterns of differentiation; processes specific to each episode of population divergence likely explain the remaining variation. The role genomic features play was reinforced by linear models identifying several genomic variables (e.g., gene densities) as significant predictors of *F_ST_* and *d_XY_* repeatability. *F_ST_* repeatability was higher among pairs that were further along the speciation continuum (i.e., more reproductively isolated) providing further insight into how genomic differentiation changes with population divergence; early stages of speciation may be dominated by positive selection that is different between pairs but becomes integrated with processes acting according to shared genomic features as speciation proceeds.

The integration of genomic data into research on population differentiation and speciation has led to the observation that genomic differentiation between closely related populations is often highly variable across the genome, with areas of elevated differentiation interspersed with areas of low differentiation (e.g., Nadeau et al. 2013; Renaut et al. 2013; Han et al. 2017; Vijay et al. 2017). One of the main conclusions from this observation is that speciation can proceed through a few focal changes and does not require divergence across the entire genome. This conclusion conforms to the genic view of speciation proposed by Wu (2001), but there is still considerable controversy concerning the factors that generate variation in estimates of genomic differentiation. This controversy has led to the development and extensive evaluation of two models.

The first model is termed divergence with gene flow (or speciation with gene flow; Nachman and Payseur 2012) and invokes both selection and gene flow to explain heterogeneous patterns of differentiation. Specifically, this model holds that divergent selection at loci involved in reproductive isolation protects some regions of the genome from gene flow, elevating an otherwise homogenized landscape of differentiation (Nosil et al. 2009; Nosil and Feder 2012). The second model proposes that selection alone can generate variation in differentiation by accelerating lineage sorting at some regions of the genome. In other words, genomic regions that do not show elevated differentiation simply continue to share ancestral polymorphism (Noor and Bennett 2009; Turner and Hahn 2010; Cruickshank and Hahn 2014). We refer to this model as selection in allopatry and note that there are variants on this model related to when selection acts (Cruickshank and Hahn 2014; Delmore et al. 2015; Irwin et al. 2016).

One common feature of both the divergence with gene flow and selection in allopatry models is that features of the local genomic landscape should contribute to variation in differentiation. For example, genomic regions with lower rates of recombination, higher rates of mutation and elevated gene densities can promote linked selection, defined as any form of selection that influences variation at nearby neutral sites (Charlesworth et al. 1993; Lohmueller et al. 2011; Charlesworth 2012; Cutter and Payseur 2013; Enard et al. 2014). Linked selection can be positive, acting on new or existing mutations (genetic hitchhiking, Maynard Smith and Haigh 1974) or purifying, removing deleterious mutations from the population (background selection, Charlesworth et al. 1993). Measures of differentiation like *F_ST_* include a term for within population variation and can be inflated by the reductions in variation that often accompany linked selection (Charlesworth 1998). Low recombination rates make it difficult for linked neutral sites to escape the effects of new mutations via recombination. Higher mutation rates and gene densities provide more targets for selection.

It was recently suggested that patterns of genomic differentiation will reflect features of the local genomic landscape more at later stages of speciation, as drift and selection at these features will take time to influence differentiation (Burri 2017). Comparative analyses examining genomic differentiation across multiple population pairs are ideal for both implicating features of the local genomic landscape in generating genomic differentiation and examining their temporal effects. For example, if genomic variables are conserved across pairs, constraints imposed by processes like linked selection in these regions should generate correlated or repeated patterns of genomic differentiation. Comparative analyses are beginning to accumulate but are often limited to a closely related group of species or populations, precluding an evaluation of temporal effects and introducing statistical nonindependence if a limited number of pairs are included (e.g., sticklebacks, Jones et al. 2012; sunflowers, Renaut et al. 2014; guppies, Fraser et al. 2015; songbirds, Irwin et al. 2016; Van Doren et al. 2017; copepods, Pereira et al. 2016). In addition, working at broader taxonomic scales may eliminate the role shared selective regimes play in generating repeatable patterns of differentiation, isolating the effects of genomic constraints.

Here, we overcome these limitations using new and archived genomic data to estimate genomic differentiation between eight pairs of birds that span a broad taxonomic range (the most recent common ancestor to them all was ∼52 MYA, http://www.timetree.org/). We look for (1) correlated patterns of genomic differentiation across these pairs (referred to as “repeatability”) and (2) an association between repeatability and the location of pairs along the speciation continuum (i.e., their level of reproductive isolation). We also (3) use linear models to implicate specific genomic features in generating repeatable patterns of genomic differentiation; these features include proxies for both recombination and mutation rates, gene density, chromosome size and proximity to chromosome ends and centromeres. We quantified the speciation continuum using hybrid zone width and genetic distance between pairs. Chromosome size, proximity to chromosome ends and centromeres may influence genomic differentiation as they have shown associations with recombination rates (Butlin 2005; Smukowski and Noor 2011). We also include linkage disequilibrium (LD) as a predictor in linear models; if linked selection is generating repeatable patterns, LD should be higher where repeatability is higher. Birds are well suited for this work as a considerable amount of information is known about speciation in this group (Price 2008) and genomic features are highly conserved across this group; birds have stable chromosome numbers, low rates of interchromosomal rearrangements, high synteny, and similar recombination landscapes (Dawson et al. 2007; Griffin et al. 2007; Backström et al. 2008; Stapley et al. 2008; Ellegren 2010; Kawakami et al. 2014; Zhang et al. 2014; Singhal et al. 2015; Kawakami et al. 2017).

Thus far we have only discussed how the local genomic landscape can affect *F_ST_*, a relative measure of differentiation that is inflated by reductions in within population variation. Many studies are beginning to include *d_XY_* in their analyses. This is an absolute measure of differentiation that does not include a term for within population variation. Under the divergence with gene flow model of speciation, those regions that contribute to reproductive isolation should have elevated *d_XY_* compared to background levels of absolute differentiation. Under the selection in allopatry model of speciation, linked selection should have no effect on *d_XY_* or reduce it compared to background levels (Nachman and Payseur 2012). The latter reductions could occur in response to recurrent linked selection in ancestral populations (which eventually results in reduced genetic distance between populations, Cruickshank et al. 2014) and/or selective sweeps of globally adaptive alleles (e.g., Delmore et al. 2015; Irwin et al. 2016). Given increasing interest in *d_XY_* and its potential to reflect the local genomic landscape, we include it in our analyses as well. All of the population pairs included in the present study occur in the temperate region where they have likely experience periods of allopatry with glacial expansions (Hewitt 2000). Accordingly, in analyses for objective 3 where we identify specific regions that show repeatable patterns we will focus on those at the bottom of the *d_XY_* distribution.

## Results

**Figure 1:**
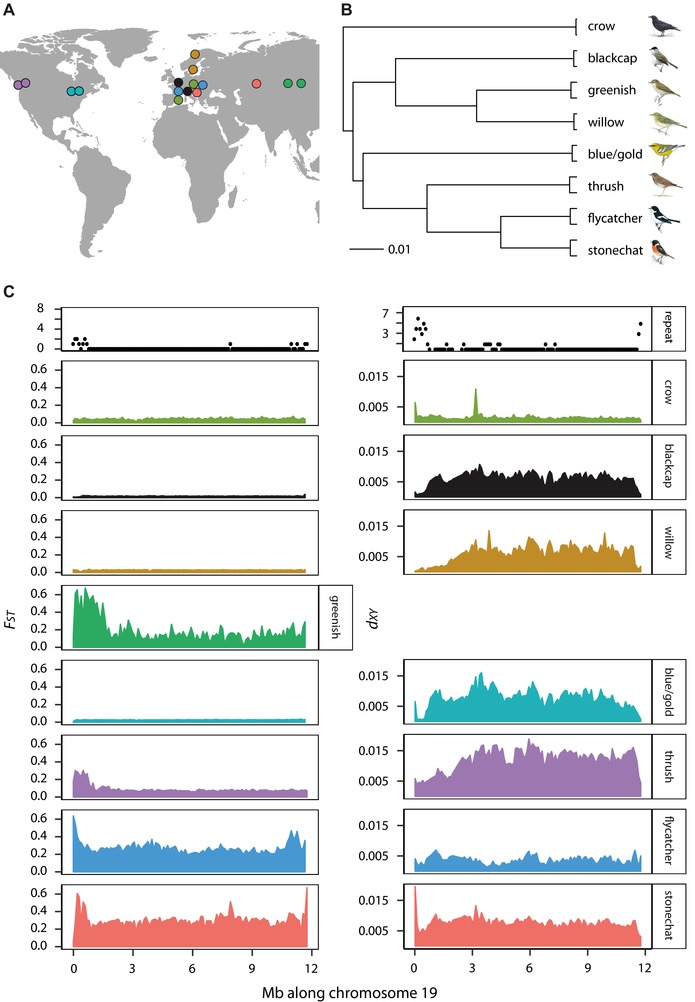
Population pairs, their (A) geographic ranges (circles as the center of sampling distributions) and (B) phylogenetic relationships (each branch has 100 bootstrapped support). Panel (C) shows windowed (100 kb) estimates of relative (*F_ST_*) and absolute (*d_XY_*) differentiation across chromosome 19 for population pairs along with repeatability at the top, measured as the number of pairs each windows as considered an outlier in (top 5 percentile of *F_ST_* distribution and bottom 5 percentile of *d_XY_* distribution; *d_XY_* not estimated for greenish warblers).

The eight pairs of birds in our study include European blackcaps (*Sylvia atricapilla*), subspecies of greenish warbler (*Phylloscopus trochiloides viridanus* and *P. t. plumbeitarsus*), subspecies of willow warbler (*Phylloscopus trochilus trochilus* and *P.t. acredula*), species of stonechat (European *Saxicola rubicola* and Siberian *S. maurus*), subspecies groups of the Swainson's thrush (coastal *Catharus ustulatus ustulatus* and inland *C. u. swainsoni*), species of flycatcher (collared *Ficedula albicollis* and pied *F. hypoleuca*), species of crow (hooded *Corvus cornix* and carrion *C. corone*), and species of wood warbler (blue‐ *Vermivora chrysoptera* and golden‐winged *V. cyanoptera* warblers; Fig. 1). To gain an overview of the relationship between these pairs, we constructed a phylogeny for the group using whole‐genome sequence data from all autosomal chromosomes (Fig. 1). The crow is the most distantly related species; all the remaining species cluster into two groups. One group includes the greenish warbler, willow warbler and blackcap while the other includes the flycatcher, stonechat, thrush, and blue/golden‐winged warblers. Disregarding sister‐pair relationships, the most closely related species are flycatchers and stonechats, and greenish and willow warblers. This topology is what we expected based on previous phylogenetic studies (e.g., Jetz et al. 2012, birdtree.org).

### REPEATABILITY IN PATTERNS OF GENOMIC DIFFERENTIATION

To estimate repeatability in patterns of genomic differentiation across pairs we organized scaffolds from each species’ reference into chromosomes using synteny with the flycatcher and estimated *F_ST_* and *d_XY_* between populations in each pair using the same 100 kb windows (Fig. 1). An initial comparison across pairs suggests that patterns may only be modestly consistent but stronger when considering *d_XY_*. We evaluated this observation by correlating windowed estimates of *F_ST_* and *d_XY_* across pairs. In accordance with our observation, correlation coefficients varied from –0.02 to 0.18 for *F_ST_* and 0.04 to 0.51 for *d_XY_* (Table 1). Squaring the highest coefficients for *F_ST_* and *d_XY_*, these results suggest that up to 3 and 26% of the variation can be explained by correlations of *F_ST_* and *d_XY_* between pairs respectively.

**Table 1:** Repeatability in genomic differentiation across population pairs of birds

	Flycatchers	Crows	Willows	Blackcaps	Greenish	Stonechats	Thrushes	Blue/gold
Flycatchers		0.17***	0.21***	0.23***		0.51***	0.11***	0.17***
Crows	0.058***		0.09***	0.14***		0.21***	0.04**	0.22***
Willows	0.020	0.028*		0.29***		0.12***	0.19***	0.24***
Blackcaps	–0.017	0.037**	0.029**			0.18***	0.44***	0.42***
Greenish	0.11***	0.054***	–0.020	0.021				
Stonechats	0.18***	0.099***	0.030**	0.045***	0.14***		0.37***	0.11***
Thrushes	0.084***	0.024**	0.12***	0.0099	0.11***	0.13***		0.21***
Blue/gold	0.0077	0.036*	0.055***	0.052***	0.027*	0.032*	0.099***	

*P*‐values corrected for multiple testing (^*^0.05, ^**^0.01, ^***^0.001).

Values are correlation coefficients comparing windowed estimates of *F_ST_* (below diagonal) and *d_XY_* (above diagonal) between each set of population pairs. *d_XY_* was not estimated for greenish warblers. For results based on outlier status and overlap values see Table S3.

We used a second method to quantify repeatability between pairs based on the overlap of outlier windows. We identified outlier windows for each pair as those in the top 5 percentile of the *F_ST_* distribution and bottom 5 percentile of the *d_XY_* distribution and compared the number of outlier windows that were shared (or overlapped) across pairs to the expected number based on the hypergeometric distribution (see Methods). Similar to results from correlations, estimates of overlap were higher and more significant for *d_XY_* (Table S3A). This was also the case when we combined strings of outlier windows into peaks, acknowledging the fact outlier windows may not be independent of one another (Table S3B).

### ASSOCIATION BETWEEN REPEATABILITY AND THE SPECIATION CONTINUUM

We looked for an association between repeatability and the speciation continuum using four different measures for the speciation continuum: hybrid zone width, the percentage of hybrids in these zones and genetic distance from both *cytb* and autosomal sequences. Starting with hybrid zone width and the percentage of hybrids, we obtained estimates for each pair from the literature and assumed reproductive isolation is greater in narrow hybrids zones with fewer hybrids (Barton and Hewitt 1985). Hybrid zone width ranged from 0 km for greenish warblers to 600 km for blue/golden‐winged warblers; the percentage of hybrids ranged from 0% for greenish warblers to 70% for willow warblers (Table S4). We transformed these values into distance matrices and compared them with the correlation matrices generated using windowed estimates *F*_ST_ and *d_XY_* above. Controlling for genetic distance between pairs, both hybrid zone width and the percentage of hybrids were negatively correlated with the with the correlation matrix based on *F_ST_* but not *d_XY_*. In other words, pairs with more narrow hybrid zones and fewer hybrids showed higher repeatability in *F_ST_* (Partial Mantel tests, hybrid zone width, *R* = –0.48, CI = –0.67‐(–0.21), *P* = 0.02; percentage of hybrids, *R* = –0.42, CI = –0.85‐(–0.0082), *P* = 0.02) but not *d_XY_* (Partial Mantel test, hybrid zone width, *R* = 0.18, CI = –0.21–0.39, *P* = 0.23; percentage of hybrids, *R* = –0.18, CI = –0.44 to 0.47, *P* = 0.45).

There are some caveats associated with using hybrid zone width and the percentage of hybrids as a proxies for reproductive isolation (e.g., differences in dispersal distance may affect estimates of hybrid zone width and there is considerable variation in how the percentage of hybrids is estimated, see Discussion). Accordingly, we reran these analyses using genetic distance between populations within each pair as a measure of reproductive isolation and assuming pairs that are more reproductively isolated from one another exhibit greater genetic distances. We estimated genetic distance using both *cytb* and autosomal sequences and similar to results using parameters from hybrid zones, we found a significant relationship between genetic distance within pairs and the correlation matrix based on *F_ST_* but not *d_XY_*; repeatability increased with genetic distance between pairs for *F_ST_* (Partial Mantel test, *cytb*, *R* = 0.41, CI = 0.23–0.53, *P* = 0.049; autosomal, *R* = 0.53, CI = 0.32–0.67, *P* = 0.009) but not *d_XY_* (Partial Mantel test, *cytb*, *R* = –0.19, CI = –0.42–0.20, *P* = 0.22; autosomal, *R* = 0.016, CI = (–0.24–0.26), *P* = 0.48).

Note, we reran these analyses replacing repeatability estimated by correlation coefficients (Table 1) with values based on the overlap of outlier windows and found similar associations (Table S3; e.g., results for *F_ST_* and hybrid zone width, *R* = –0.41, CI = –0.72‐(–0.15), *P* = 0.03; *cytb*, *R* = 0.33, CI = 0.05–0.52, *P* = 0.05; autosomal, *R* = 0.41, CI = 0.22–0.59, *P* = 0.006). This is an important finding, as it suggests the associations we documented are not related to the fact that there is a greater range of *F_ST_* values at later stages of speciation.

### GENOMIC FEATURES OR PROCESSES AS PREDICTORS OF REPEATABILITY

The repeated patterns of *F_ST_* and *d_XY_* we documented above suggest that variation in genome‐wide estimates of differentiation is influenced by conserved features of the local genomic landscape. We used generalized linear models (GLMs) to evaluate the role‐specific genomic features play in generating these patterns. Repeatability was quantified for each window as the number of pairs in which the window was considered an outlier (recall outlier status was determined using the top 5 percentile of the *F_ST_* distribution and bottom 5 percentile of the *d_XY_* distribution). Separate GLMs were run for each species pair using seven predictor variables (estimated for each pair separately): the proportion of GC bases (proxy for recombination rate), synonymous mutation rate (*d_s_*; proxy for mutation rate), gene count, LD, and three variables related to where windows are located in the genome (micro‐ or macrochromosomes [chromosome size], proximity to chromosome ends, and centromeres).

**Figure 2:**
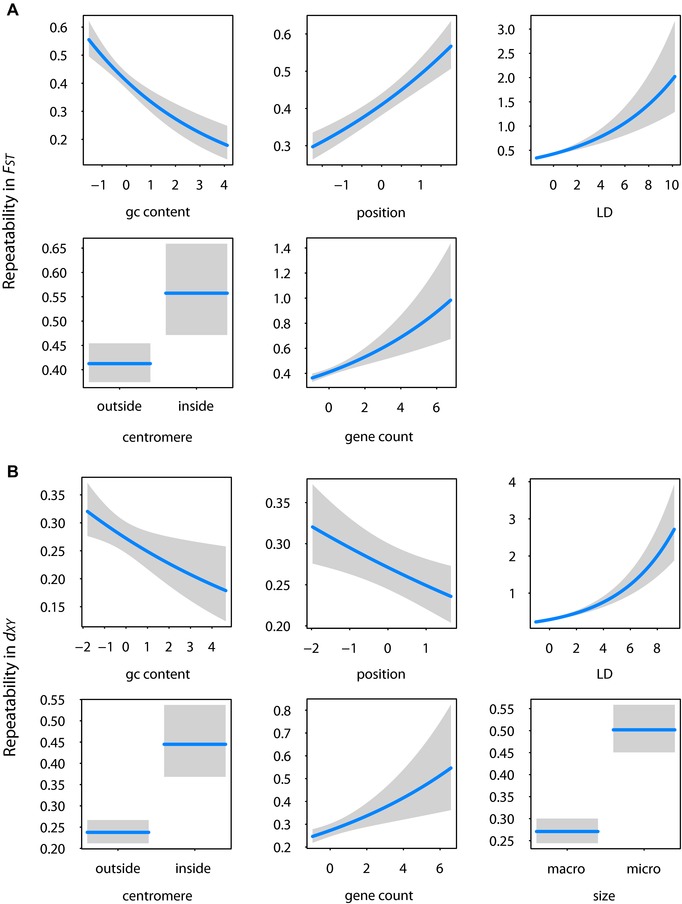
Results from GLMs examining the relationship between repeatability and predictor variables related to features of the local genomic landscape. Relationships shown are limited to significant predictor variables and results from blackcaps (results for nonsignificant predictor variables and the remaining population pairs and can be found in Table S5). Repeatability is estimated as the number of pairs each window was considered an outlier in (outliers are windows in the top 5 percentile of each species pairs’ distribution for FST [A] and bottom 5 percentile for dXY [B]). Correlation coefficients for full models are 0.17 for FST and 0.27 for dXY. Parameter estimates are as follows: FST, GC content –0.20 [±0.04, *P* < 0.001], position 0.19 [±0.03, *P* < 0.001], gene count 0.13 [±0.03, *P* < 0.001], linkage disequilibrium 0.15 [±0.02, *P* < 0.001], centromere 0.32 [±0.09, *P* < 0.001]; dXY: GC content –0.09 [±0.04, *P* = 0.01], position –0.08 [±0.03, *P* < 0.001], gene count 0.11 [±0.03, *P* < 0.001], size 0.62 (±0.08, *P* < 0.001], linkage disequilibrium 0.24 [±0.02, *P* < 0.001], centromere 0.63 [±0.11, *P* < 0.001]). Each predictor is scaled and their effects are plotted with other variables held at their medians. A positive association with position indicates increased repeatability at the center of chromosomes.

Results from these GLMs can be found in Figure 2 (with blackcaps as predictor) and Table S5 (for each case as predictor). GC content, gene density, LD, and proximity to both chromosome ends and centromeres were significant predictors of repeatability in *F_ST_* across all species pairs. Each of these variables was positive predictors of repeatability, except GC content that was negative. In the case of position, this means that windows near the center of chromosomes showed higher repeatability. Linkage disequilibrium, proximity to centromeres, and chromosome size were significant predictors of repeatability in *d_XY_* across all species pairs. In the case of chromosome size, this means that windows on microchromosomes show more consistent patterns. GC content, proximity to chromosome ends, gene density, and *d_s_* were also significant predictors of repeatability in *d_XY_* but not for all species pairs.

Note that while the predictor variables used in these models are species‐specific (e.g., gene density estimate for each pair separately) the response variable is the same–‐a summary variable quantifying repeatability across species pairs. Accordingly, results from these models are not entirely independent.

## Discussion

We used genomic data from eight population pairs of birds that span a broad taxonomic scale to study the contribution of local genomic features to variation in genome‐wide estimates of differentiation. We rely on the fact genomic features are conserved across birds to draw inferences from our analyses and discuss these findings below, including the potential temporal effect these features can have of genomic differentiation.

Our first objective was to determine if patterns of genomic differentiation were correlated (or repeated) across population pairs. Our results suggest that up to 3% of the variation in *F_ST_* and 26% of the variation in *d_XY_* can be explained by correlations across pairs. Shared features of the genomic landscape likely contribute to these correlations. For example, linked selection (positive [genetic hitchhiking] or purifying [background selection]) is influenced by genomic features and tends to reduce within population variation, inflating *F_ST_*. Many of these genomic features are conserved across birds (Dawson et al. 2007; Griffin et al. 2007; Backström et al. 2008; Stapley et al. 2008; Ellegren 2010; Kawakami et al. 2014; Zhang et al. 2014; Singhal et al. 2015; Kawakami et al. 2017) and likely generated the repeatable patterns we observed. In support of this suggestion, recombination rates (approximated by GC content) and gene densities, two genomic features that are preserved across birds and influence linked selection were consistent predictors of repeatability in *F_ST_*. Genetic drift may also contribute to the correlations we documented. For example, drift in a low recombination region can cause it to show consistently high or low *F_ST_*. Nevertheless, drift is generally expected to reduce genetic diversity genome‐wide. Note that the amount of variation explained by correlations across pairs is not as high as other studies (e.g., 49–77% of the variation in *F_ST_* explained by correlations across subspecies pairs of greenish warblers in Irwin et al. 2016). Nevertheless, most of these studies focus on pairs that are much more closely related than those in the present study, such that pairs share more genomic features and are subject to similar selective forces.

Our second objective was to determine if there was a positive association between repeatability and the location of pairs along the speciation continuum. We measured the speciation continuum using hybrid zone width, the proportion of hybrids in these zones and genetic distance and found that pairs with narrower hybrid zones and greater genetic distances exhibited more similar patterns of *F_ST_*. The latent effects of drift and selection may explain this pattern. Specifically, when population divergence begins, allele frequencies will be roughly equal and *F_ST_* will be close to zero. Drift and selection will start acting on standing genetic variation and any new mutations that arise. The effects of these processes, especially drift and background selection, may take time to accumulate (Burri 2017). Accordingly, positive selection and genetic hitchhiking may be the primary forces affecting differentiation early in speciation. If the selective context of speciation is different for the pairs under study, this should lead to less repeatable (or correlated) patterns of differentiation. As speciation proceeds, drift and background selection will begin to affect differentiation as well and, combined with positive selection and genetic hitchhiking, these processes could result in the landscape of differentiation reflecting genomic features more directly. This scenario was described by Burri (2017) and is in line with recent work showing that linked selection (positive or purifying in nature) may generate repeated patterns of differentiation at longer time scales (Phung et al. 2016; Dutoit et al. 2017; Van Doren et al. 2017; Vijay et al. 2017). Note that the beginning stages of speciation may be less repeatable even without different selective pressures. For example, there may be more than one way to respond to selection and the chance positive selection affects the same genomic region may be low.

Our findings related to the speciation continuum will require additional study. To begin with, we used Partial Mantel tests for these analyses that may be prone to Type I errors (Harmon and Glor 2010, but see Diniz‐Filho et al. 2013). Alternatives exist (e.g., Redundancy Analyses or correlograms) but require larger sample sizes. Our use of hybrid zone width to quantify the speciation continuum also assumes that dispersal distance is the same across all pairs. This is a common assumption among songbirds as it is difficult to obtain unbiased estimates of dispersal for this group that is based on large sample sizes. Analyses using the percentage of hybrids are not affected by dispersal distance but are associated with another set of assumptions, including that each study had the same resolution to identify hybrids and used similar sampling strategies. Regardless, the association we documented between repeatability and the speciation continuum is intriguing and was documented using not only parameters from hybrid zones but also genetic distance between populations in each pair.

Thus far we have focused mainly on results for *F_ST_*; results for *d_XY_* require careful explanation. Concerning the repeatable patterns we documented (first objective, up to 26% of the variation in *d_XY_* explained by correlations across pairs), at the outset we argued that speciation in the pairs we studied would have been punctuated with periods of allopatry as all pairs occur in the temperate region where glacial advances would have isolated populations in different refugia (Hewitt 2000). Under this scenario (i.e., without gene flow), *d_XY_* should reflect the amount of sequence divergence that has been acquired since populations split (along with variation that existed in the common ancestor) and linked selection should have no effect on *d_XY_* or reduce it (e.g., if recurrent linked selection in ancestral populations removes variation from populations prior to their split; Nachman and Payseur 2012; Cruickshank and Hahn 2014). Consistent with the above scenario, patterns of *d_XY_* were repeatable across pairs and several genomic features including gene densities and chromosome size predicted repeatable patterns at the bottom of the *d_XY_* distribution. Nevertheless, it is important to note that during periods of secondary contact gene flow will reduce *d_XY_* in some regions, generating peaks of differentiation. Accordingly, some of the repeatable patterns we documented may also be related to elevated *d_XY_* (Nachman and Payseur 2012).

Continuing with *d_XY_* repeatability (i.e., results for the first objective), correlation coefficients were higher for *d_XY_* than *F_ST_*, with the highest correlation coefficient for *d_XY_* being more than twice that for *F_ST_* (0.18 vs 0.51). This finding could be related to the fact that *d_XY_* reflects processes that have accumulated over multiple speciation events (see below for additional explanation) while *F_ST_* mainly reflects processes in extant populations. Accordingly, if population pairs are sampled too early in speciation, *F_ST_* may not reflect local genomic features yet as it will take time to accumulate (Burri 2017). This suggestion follows from the argument described above about the latent effects of drift and background selection. Nevertheless, additional explanations for increased *d_XY_* repeatability are also possible. For example, *d_XY_* shows a strong relationship with mutation rates (Geneva et al. 2015; Rosenzweig et al. 2016). Accordingly, much of the pattern we documented may be related solely to variation in mutation rates. It is also important to note that *d_XY_* is estimated using far more sites than *F_ST_* (variant and invariant for *d_XY_* vs just variant for *F_ST_*). Accordingly, these estimates may be more precise, leading to stronger correlation coefficients.

Finally, while we found a correlation between the speciation continuum and repeatability in *F_ST_* (second objective) we did not document this association for *d_XY_*. Again, this finding may be related to the fact that *d_XY_* reflects processes that have accumulated over multiple speciation events while *F_ST_* mainly reflects process in extant populations, including speciation. For example, if the recombination landscape has remained the same for millions of years, recurrent linked selection in areas of low recombination has likely been reducing variation over the same time period and these reductions will be passed down over speciation events (Burri 2017). Under this scenario, it will not matter what stage of differentiation the population pairs under study are at, these reductions will be reflected in estimates of *d_XY_*. As we have already discussed, there are situations where *d_XY_* will reflect processes in extant populations (especially if gene flow is occurring) but the underlying effect of ancestral diversity appears to override any effect these processes have on *d_XY_* repeatability and the speciation continuum.

We documented modest but significant repeatability in relative and absolute differentiation across eight population pairs of birds and showed that several genomic features predicted this repeatability. As genomic features are conserved across birds, these results suggest that at least moderate amounts of variation in genome‐wide differentiation can be attributed to processes acting at genomic features, including linked selection that may derive from both positive and purifying selection. A considerable amount of the remaining variation in genomic differentiation is likely related to processes specific to each episode of population divergence. This is especially true for pairs early in the process of speciation, as our observation that repeatability increases with the location of pairs along the speciation continuum suggests processes acting at shared genomic features become more important later in this processes. To the best of our knowledge, this is the first empirical support for a temporal role of genomic features in structuring genomic differentiation and we encourage future studies incorporating additional pairs to study this association and the genetics of speciation. Studies focused on a single system and points in time provide only a snapshot of this extensive and often prolonged process.

## Methods

### STUDY SPECIES AND DATASETS

We searched the Sequence Read Archive (https://www.ncbi.nlm.nih.gov/sra) and European Nucleotide Archive (http://www.ebi.ac.uk/ena) for genomic data collected from birds. We limited our search to species for which a draft reference genome had been assembled for the target species or one that was closely related. This search resulted in the inclusion of eight pairs (Table S1). The only pair we did not have a reference genome for was the *Vermivora* warblers but a reference for the closely related yellow‐rumped warblers (*Setophaga coronata*) is available and was used in the original publication for these data (Toews et al. 2016). All pairs are from the order Passeriformes (perching birds or songbirds) and breed in temperate regions (Fig. 1).

### GENERATING CONSENSUS REFERENCE GENOMES

The reference genomes we acquired were all assembled into scaffolds, except the collared flycatcher's genome, which was organized into chromosomes based on linkage map for the species and synteny with zebra finch (Ellegren et al. 2012). Accordingly, we used this genome to ensure all windows compared across species were orthologous. To maintain chromosomal synteny, we aligned the scaffolds of each genome individually against the flycatcher genome with SatsumaSynteny (default parameters; Grabherr et al. 2010). We then used bash scripts to parse the output, obtaining information on the order and orientation of query scaffolds and conducted a final alignment with the LASTZ plugin in Geneious (Harris 2007; Kearse et al. 2012). We merged these scaffolds into pseudochromosomes by calling the query base where alignments occurred and Ns in the presence of a gap. Details on consensus genome coverage can be found in Table S2.

### CONSTRUCTING PHYLOGENETIC NETWORK

We used ANGSD (Korneliussen et al. 2014) to obtain consensus fasta sequences for populations from each species pair (‐doFasta 2 ‐doCounts 1 ‐minQ 20 ‐setMinDepth 10) and IQ‐TREE (Nguyen et al. 2016) to construct a maximum‐likelihood tree from these sequences. Patristic distance between pairs was estimated using this tree using the cophenetic.phylo function from the R package “ape.” To compare relative divergence across species pairs we used the chronos function from the same R package, using the default of correlated rate model and generating an ultrametric tree.

### ESTIMATING DIFFERENTIATION

We focused on SNPs for the present study and used a common reference‐based bioinformatics pipeline to call them. Details can be found in Delmore et al. (2015, 2016). Briefly, we trimmed reads with trimmomatic (TRAILING:3 SLIDINGWINDOW:4:10 MINLEN:30) and aligned them to consensus genomes using bwa *mem* (Li and Durbin 2009) using default settings. We then used GATK (McKenna et al. 2010) and picardtools (http://broadinstitute.github.io/picard) to identify and realign reads around indels (*RealignerTargetCreator*, *IndelRealigner*, default settings) and removed duplicates (*MarkDuplicates*, default settings) for all datasets except greenish warbler that consisted of GBS data.

We used two estimates of differentiation in our study: *F_ST_* and *d_XY_*. We estimated *F_ST_* for datasets comprised of individuals using ANGSD, estimating site frequency spectrums for each population separately (‐dosaf 1, ‐gl 1, ‐remove_bads, ‐unique_only, ‐minMapQ 20, ‐minQ 20, ‐only_proper_pairs 1, ‐trim 0) and using these to obtain joint frequencies spectrums for population pairs. These joint frequency spectrums were then used as priors for allele frequencies at each site to estimate *F_ST_*. For datasets comprised of pools we estimated *F_ST_* using Popoolation2 (Kofler et al. 2011; ‐min‐coverage 30 for Swainson's thrushes and 10 for stonechats, ‐min‐count 3, ‐minq 20). We summarized *F_ST_* into windows of 100 kb and limited analyses to windows with data from all pairs. We excluded the Z chromosome from all analyses as some of the pairs included females where systematic biases related to coverage could affect estimates of differentiation.

We estimated *d_XY_* for datasets comprised of individuals using ANGSD as well. First, we estimated allele frequencies at each SNP for both populations of each pair combined ‐doMajorMinor 4, ‐doMaf 2, ‐gl 1, ‐doCounts 1, ‐remove_bads, ‐unique_only, ‐minMapQ 20, ‐minQ 20, ‐only_proper_pairs 1, ‐trim 0, ‐SNP_pval 1e‐6). We then reran the program by population using only the SNPs that passed the previous step, to ensure SNPs fixed in one population were not lost. Once we had these allele frequencies, we estimate *d_XY_* at each SNP using a script provided with ANGSD (https://github.com/mfumagalli/ngsPopGen//scripts/calcDxy.R) and as (p1*(1−p2))+(p2*(1−p1)) where p is the allele frequency of a given allele in populations 1 and 2, respectively and averaged these values in the same 100 kb windows used for *F_ST_*. Estimates of *d_XY_* by SNP have to be normalized by the number of sites (variant and invariant) in a window. We obtained these values using ANGSD to estimate read depth at all sites (‐doCounts 1, ‐dumpCounts 1, ‐remove_bads, ‐unique_only, ‐minMapQ 20, ‐minQ 20, ‐only_proper_pairs 1, ‐trim 0) and excluded sites with coverage less than three times the sample size and more than three times the average coverage to ensure roughly three reads per individual and exclude sites that may have mapping problems resulting from copy number variants. Analyses were limited to windows with data from all pairs and windows with more than 5000 callable sites, as *d_XY_* can be highly variable with small sample sizes and coverage (e.g., Clarkson et al. 2014). This filter precluded the use of greenish warblers in analyses of *d_XY_* as data for this pair were derived from reduce‐representation sequencing and did not have high coverage in windows of 100 kb. This was not a problem for the original publication (Irwin et al. 2016) as windows were defined by SNPs rather than base pairs.

For stonechats and thrushes, for which we used pooled sequencing data, we calculated *d_XY_* with a custom script. We excluded sites with coverage below 10 for stonechats and below 30 for thrushes. We estimated *d_XY_* by multiplying allele frequencies for each base as above and averaging across sufficiently covered bases in each window.

### ESTIMATING REPEATABILITY IN PATTERNS OF GENOMIC DIFFERENTIATION

We estimated overall repeatability between pairs by correlating windowed‐estimates of differentiation across pairs. We also estimated repeatability using information on outlier status and overlap. Specifically, we identified outlier windows for each species pairs as those above the top 5% quantile for *F_ST_* and below the bottom 5% quantile for *d_XY_*. For each comparison across pairs (e.g., flycatcher to stonechat, flycatcher to greenish warbler, etc.), we counted the number of outlier windows that were shared (or overlapped) and compared this to the expected number of overlapping windows using the hypergeometric distribution, assuming that each window had an equal probability of being considered an outlier. We used these values (observed and expected) to calculate z scores for each comparison and calculated one‐sided *P*‐values (i.e., the probability of obtaining an overlap value as extreme or more extreme than our observed value). Z scores are effect sizes that correspond to the number of standard deviations above the average expectation in each comparison, allowing for direct comparison across studies. Note that it is also possible that the outlier windows we identified are not independent of one another. Accordingly, we reran this analysis modifying our overlap approach by combining outlier windows into peaks if they occurred next to one another and using the number of peaks as our estimate of overlap. We used a permutation test to quantify the significance of these values, holding the number and size of outlier regions constant while randomly permuting their location 1000 times and calculating one‐sided *P*‐values again.

### PLACING PAIRS ALONG THE SPECIATION CONTINUUM

We used four methods to place pairs along the speciation continuum (i.e., to quantify the level of reproductive isolation), starting with the width of hybrid zones and percentage of hybrids in these zones, which we obtained from the literature. The more narrow a zone and the fewer hybrids present the higher the reproductive isolation (Barton and Hewitt 1985; Moore and Dolbeer 1989; Paradis et al. 1998). If hybridization is extremely rare (e.g., with the greenish warblers), we set the width as zero. We also used genetic distance within pairs, which we obtained by aligning *ctyb* sequences (downloaded from NCBI https://www.ncbi.nlm.nih.gov/) using MEGA as *p*‐distance (the proportion of nucleotide sites that differ between groups) and the distance matrix generated by IQTREE for autosomal chromosome alignments (see “Constructing phylogenetic network”). We used Mantel tests to compare distance matrices quantifying the speciation continuum with distance matrices quantifying repeatability. We accounted for small sample sizes in these tests by using permutation tests to quantify significance and the non‐parametric Spearman rank correlation coefficient.

### MEASURING GENOMIC FEATURES AND PROCESSES AND THEIR EFFECT ON REPEATABILITY

We looked at the relationship between repeatability and seven structural features of the genome: recombination rate, mutation rate, gene density, chromosome size, proximity to chromosome ends, and centromeres and linkage disequilibrium. We estimated these features for each species and ran separate generalized linear models (GLMs) with repeatability as the response variable with a Poisson distribution for each species. These models were run with the glm function in base R and the ANOVA function was used to evaluate the significance of each predictor variable. We visualized results with the “visreg” package and estimated correlation coefficients (and confidence intervals) for each model by regressing observed repeatability to repeatability predicted by each model.

We used GC content as a proxy for recombination. Recombination affects the patterns of local base composition via the unbalanced transmission of “strong” (GC) over “weak” (AT) alleles at double‐strand breaks (Mugal et al. 2015). This process is termed GC‐biased gene conversion and direct support was recently presented in birds (Smeds et al. 2016). Positive correlations between recombination and GC content have also been documented in birds (Kawakami et al. 2014; Burri et al. 2015). Synonymous mutations occur in the exon of genes but have no effect on the sequence of amino acids. The use of *d_s_* for mutation rate analysis assumes these sites do not experience selection (Eyre‐Walker and Keightley 1999). We used a phylogenetic framework to obtain these estimates; details can be found in the Supplementary Methods. Briefly, we annotated each consensus genome with MAKER and identified potential homologues for high quality transcripts (AED < 0.05) using a Blastn search against all transcripts from the flycatcher (flycatcher was searched against zebra finch). We aligned codons from each pair of sequences using PRANK (http://wasabiapp.org/software/prank) and calculated *d_N_/d_s_* with PAML v4.8. All *d_N_/d_s_* calculations were performed pairwise, comparing all the species with the flycatcher and this in turn, compared to zebra finch. Estimates of *d_s_* were extracted for GLMs. We used PLINK 1.9 (Chang et al. 2015) to estimate linkage disequilibrium for one population from each pair (the same population for which we had a reference genome), as the squared correlation coefficient (*r^2^*) between pairs of SNPs. SNPs were output from ANGSD using the same filters described above for *F_ST_* and *d_XY_* and including an additional filter for minor allele frequency, requiring SNPs have minor allele frequencies greater than 0.05. PLINK was run with the command line with the command line “–ld‐window 100 –ld‐window‐kb 100 –ld‐window‐r2 0” to limit the analyses to SNPs with fewer than 100 variants between them and no more than 100 kilobases apart and report pairs with *r^2^* values below 0.2 as well. We determined the midpoints for all SNP pairs, binned them into the same 100 kb windows used for *F_ST_* and *d_XY_* and calculated average values for each window.

Avian genomes are composed of micro‐ and macrochromosomes. We considered microchromosomes those that are less than 20 Mb (Ellegren 2013) and macrochromosomes those that are greater than 40 Mb. We identified the position of each window along the chromosome by dividing chromosomes in half and generating a variable that increased by a value of 1 for each window from the end of the chromosome to its center. We standardized this measure by dividing these values by half the number of windows on each chromosome, so values increased to 1 at the center of chromosomes. We inferred the location of centromeres using methods employed by Ellegren et al. (2012) and Delmore et al. (2015). Specifically, we identified FISH probes from Warren *et al*. (2010) on either side of centromeres in the zebra finch genome and used NCBI's blastn (Altschul et al. 1990) to find their location in our genome. We considered sequences between FISH probes as “centromeric regions” and note this method only gives us a rough approximation for the location of centromeres.

Associate Editor: Z. Gompert

## Supporting information


**Table S1**. Datasets included in the present study.
**Table S2**. Coverage of consensus genomes.
**Table S3**. Repeatability measured using outlier status and overlap values.
**Table S4**. Data used to measure the speciation continuum.

**Table S5**. Results from GLMs examining the relationship between repeatability and predictor variables related to genomic features.

## References

[evl346-bib-0001] Altschul, S. F. , W. Gish , W. Miller , E. W. Myers , and D. J. Lipman . 1990 Basic local alignment search tool. J. Mol. Biol. 215:403–410. 2231712 10.1016/S0022-2836(05)80360-2

[evl346-bib-0002] Backström, N. , N. Karaiskou , E. H. Leder , L. Gustafsson , C. R. Primmer , A. Qvarnström , et al. 2008 A gene‐based genetic linkage map of the collared flycatcher (*Ficedula albicollis*) reveals extensive synteny and gene‐order conservation during 100 million years of avian evolution. Genetics 179:1479–1495. 18562642 10.1534/genetics.108.088195 PMC2475748

[evl346-bib-0003] Barton, N. H. , and G. M. Hewitt . 1985 Analysis of hybrid zones. Annu. Rev. Ecol. Syst. 16:113–148.

[evl346-bib-0004] Burri, R. 2017 Interpreting differentiation landscapes in the light of long‐term linked selection. Evol. Lett. 1:118–131.

[evl346-bib-0005] Burri, R. , A. Nater , T. Kawakami , C. F. Mugal , P. I. Olason , L. Smeds , et al. 2015 Linked selection and recombination rate variation drive the evolution of the genomic landscape of differentiation across the speciation continuum of *Ficedula flycatchers* . Genome Res. 25:1656–1665. 26355005 10.1101/gr.196485.115 PMC4617962

[evl346-bib-0006] Butlin, R. K. 2005 Recombination and speciation. Mol. Ecol. 14:2621–2635. 10.1111/j.1365-294X.2005.02617.x 16029465

[evl346-bib-0007] Charlesworth, B. 1998 Measures of divergence between populations and the effect of forces that reduce variability. Mol. Biol. Evol. 15:538–543. 9580982 10.1093/oxfordjournals.molbev.a025953

[evl346-bib-0008] Charlesworth, B. 2009 Effective population size and patterns of molecular evolution and variation. Nat. Rev. Genet. 10:195–205. 19204717 10.1038/nrg2526

[evl346-bib-0009] Charlesworth, B. 2012 The role of background selection in shaping patterns of molecular evolution and variation: evidence from variability on the *Drosophila* X chromosome. Genetics 191:233–246. 22377629 10.1534/genetics.111.138073 PMC3338263

[evl346-bib-0010] Charlesworth, B. , M. T. Morgan , and D. Charlesworth . 1993 The effect of deleterious mutations on neutral molecular variation. Genetics 134:1289–1303. 8375663 10.1093/genetics/134.4.1289 PMC1205596

[evl346-bib-0011] Clarkson, C. S. , D. Weetman , J. Essandoh , A. E. Yawson , G. Maslen , M. Manske , et al. 2014 Adaptive introgression between *Anopheles* sibling species eliminates a major genomic island but not reproductive isolation. Nat. Commun. 5(ncomms5): 248. 10.1038/ncomms5248 PMC4086683 24963649

[evl346-bib-0012] Cruickshank, T. E. , and M. W. Hahn . 2014 Reanalysis suggests that genomic islands of speciation are due to reduced diversity, not reduced gene flow. Mol. Ecol. 23:3133–3157. 24845075 10.1111/mec.12796

[evl346-bib-0013] Cutter, A. D. , and B. A. Payseur . 2013 Genomic signatures of selection at linked sites: unifying the disparity among species. Nat. Rev. Genet. 14:262–274. 23478346 10.1038/nrg3425 PMC4066956

[evl346-bib-0014] Dawson, D. A. , M. Åkesson , T. Burke , J. M. Pemberton , J. Slate , and B. Hansson . 2007 Gene order and recombination rate in homologous chromosome regions of the chicken and a passerine bird. Mol. Biol. Evol. 24:1537–1552. 17434902 10.1093/molbev/msm071

[evl346-bib-0015] Delmore, K. E. , S. Hübner , N. C. Kane , R. Schuster , R. L. Andrew , F. Câmara , et al. 2015 Genomic analysis of a migratory divide reveals candidate genes for migration and implicates selective sweeps in generating islands of differentiation. Mol. Ecol. 24:1873–1888. 25808860 10.1111/mec.13150

[evl346-bib-0016] Delmore, K. E. , D. P. L. Toews , R. R. Germain , G. L. Owens , and D. E. Irwin . 2016 The genetics of seasonal migration and plumage color. Curr. Biol. 26:2167–2173. 27476599 10.1016/j.cub.2016.06.015

[evl346-bib-0017] Diniz‐Filho, J. A. F. , T. N. Soares , J. S. Lima , R. Dobrovolski , V. L. Landeiro , M. P. de Campos Telles , et al. 2013 Mantel test in population genetics. Genet. Mol. Biol. 36:475–485. 24385847 10.1590/S1415-47572013000400002 PMC3873175

[evl346-bib-0018] Dutoit, L. , N. Vijay , C. F. Mugal , C. M. Bossu , R. Burri , J. Wolf , et al. 2017 Covariation in levels of nucleotide diversity in homologous regions of the avian genome long after completion of lineage sorting. Proc. R. Soc. B. 284: 20162756. 10.1098/rspb.2016.2756 PMC5326536 28202815

[evl346-bib-0019] Ellegren, H. 2010 Evolutionary stasis: the stable chromosomes of birds. Trends Ecol. Evol. 25:283–291. 20363047 10.1016/j.tree.2009.12.004

[evl346-bib-0020] Ellegren, H. 2013 The evolutionary genomics of birds. Annu. Rev. Ecol. Evol. Syst. 44:239–259.

[evl346-bib-0021] Ellegren, H. , L. Smeds , R. Burri , P. I. Olason , N. Backström , T. Kawakami , et al. 2012 The genomic landscape of species divergence in *Ficedula flycatchers* . Nature 491:756–760. 23103876 10.1038/nature11584

[evl346-bib-0022] Enard, D. , P. W. Messer , and D. A. Petrov . 2014 Genome‐wide signals of positive selection in human evolution. Genome Res. 24:885–895. 24619126 10.1101/gr.164822.113 PMC4032853

[evl346-bib-0023] Eyre‐Walker, A. , and P. D. Keightley . 1999 High genomic deleterious mutation rates in hominids. Nature 397:344–347. 9950425 10.1038/16915

[evl346-bib-0024] Eyre‐Walker, A. , and P. D. Keightley . 2007 The distribution of fitness effects of new mutations. Nat. Rev. Genet. 8:610–618. 17637733 10.1038/nrg2146

[evl346-bib-0025] Fraser, B. A. , A. Künstner , D. N. Reznick , C. Dreyer , and D. Weigel . 2015 Population genomics of natural and experimental populations of guppies (*Poecilia reticulata*). Mol. Ecol. 24:389–408. 25444454 10.1111/mec.13022

[evl346-bib-0026] Geneva, A. J. , C. A. Muirhead , S. B. Kingan , and D. Garrigan . 2015 A new method to scan genomes for introgression in a secondary contact model. PloS One 10:e0118621. 25874895 10.1371/journal.pone.0118621 PMC4396994

[evl346-bib-0027] Grabherr, M. G. , P. Russell , M. Meyer , E. Mauceli , J. Alföldi , F. Di Palma , et al. 2010 Genome‐wide synteny through highly sensitive sequence alignment: Satsuma. Bioinformatics 26:1145–1151. 20208069 10.1093/bioinformatics/btq102 PMC2859124

[evl346-bib-0028] Griffin, D. K. , L. B. W. Robertson , H. G. Tempest , and B. M. Skinner . 2007 The evolution of the avian genome as revealed by comparative molecular cytogenetics. Cytogenet. Genome Res. 117:64–77. 17675846 10.1159/000103166

[evl346-bib-0029] Han, F. , S. Lamichhaney , B. R. Grant , P. R. Grant , L. Andersson , and M. T. Webster . 2017 Gene flow, ancient polymorphism, and ecological adaptation shape the genomic landscape of divergence among Darwin's finches. Genome Res. 27:1004–1015. 28442558 10.1101/gr.212522.116 PMC5453315

[evl346-bib-0030] Harmon, L. J. , and R. E. Glor . 2010 Poor statistical performance of the Mantel test in phylogenetic comparative analyses. Evol. Int. J. Org. Evol. 64:2173–2178. 10.1111/j.1558-5646.2010.00973.x 20163450

[evl346-bib-0031] Harris, R. S. 2007 Improved pairwise alignment of genomic DNA. Pennsylvania State University.

[evl346-bib-0032] Hewitt, G. 2000 The genetic legacy of the Quaternary ice ages. Nature 405:907–913. 10879524 10.1038/35016000

[evl346-bib-0033] Irwin, D. E. , M. Alcaide , K. E. Delmore , J. H. Irwin , and G. L. Owens . 2016 Recurrent selection explains parallel evolution of genomic regions of high relative but low absolute differentiation in a ring species. Mol. Ecol. 25:4488–4507. 27484941 10.1111/mec.13792

[evl346-bib-0034] Jetz, W. , G. H. Thomas , J. B. Joy , K. Hartmann , and A. O. Mooers . 2012 The global diversity of birds in space and time. Nature 491:444–448. 23123857 10.1038/nature11631

[evl346-bib-0035] Jones, F. C. , M. G. Grabherr , Y. F. Chan , P. Russell , E. Mauceli , J. Johnson , et al. 2012 The genomic basis of adaptive evolution in threespine sticklebacks. Nature 484:55–61. 22481358 10.1038/nature10944 PMC3322419

[evl346-bib-0036] Kawakami, T. , C. F. Mugal , A. Suh , A. Nater , R. Burri , L. Smeds , et al. 2017 Whole‐genome patterns of linkage disequilibrium across flycatcher populations clarify the causes and consequences of fine‐scale recombination rate variation in birds. Mol. Ecol. 26:4158–4172. 28597534 10.1111/mec.14197

[evl346-bib-0037] Kawakami, T. , L. Smeds , N. Backström , A. Husby , A. Qvarnström , C. F. Mugal , et al. 2014 A high‐density linkage map enables a second‐generation collared flycatcher genome assembly and reveals the patterns of avian recombination rate variation and chromosomal evolution. Mol. Ecol. 23:4035–4058. 24863701 10.1111/mec.12810 PMC4149781

[evl346-bib-0038] Kearse, M. , R. Moir , A. Wilson , S. Stones‐Havas , M. Cheung , S. Sturrock , et al. 2012 Geneious basic: an integrated and extendable desktop software platform for the organization and analysis of sequence data. Bioinformatics 28:1647–1649. 22543367 10.1093/bioinformatics/bts199 PMC3371832

[evl346-bib-0039] Kofler, R. , R. V. Pandey , and C. Schlötterer . 2011 PoPoolation2: identifying differentiation between populations using sequencing of pooled DNA samples (Pool‐Seq). Bioinformatics 27:3435–3436. 22025480 10.1093/bioinformatics/btr589 PMC3232374

[evl346-bib-0040] Korneliussen, T. S. , A. Albrechtsen , and R. Nielsen . 2014 ANGSD: analysis of next generation sequencing data. BMC Bioinformatics 15:1. 25420514 10.1186/s12859-014-0356-4 PMC4248462

[evl346-bib-0041] Li, H. , and R. Durbin . 2009 Fast and accurate short read alignment with Burrows–Wheeler transform. Bioinformatics 25:1754–1760. 19451168 10.1093/bioinformatics/btp324 PMC2705234

[evl346-bib-0042] Lohmueller, K. E. , A. Albrechtsen , Y. Li , S. Y. Kim , T. Korneliussen , N. Vinckenbosch , et al. 2011 Natural selection affects multiple aspects of genetic variation at putatively neutral sites across the human genome. PLOS Genet. 7:e1002326. 22022285 10.1371/journal.pgen.1002326 PMC3192825

[evl346-bib-0043] Maynard Smith, J. M. , and J. Haigh . 1974 The hitch‐hiking effect of a favourable gene. Genet. Res. 23:23–35. 4407212

[evl346-bib-0044] McKenna, A. , M. Hanna , E. Banks , A. Sivachenko , K. Cibulskis , A. Kernytsky , et al. 2010 The genome analysis toolkit: a MapReduce framework for analyzing next‐generation DNA sequencing data. Genome Res. 20:1297–1303. 20644199 10.1101/gr.107524.110 PMC2928508

[evl346-bib-0045] Moore, W. S. , and R. A. Dolbeer . 1989 The use of banding recovery data to estimate dispersal rates and gene flow in avian species: case studies in the red‐winged blackbird and common grackle. Condor 91:242–253.

[evl346-bib-0046] Mugal, C. F. , C. C. Weber , and H. Ellegren . 2015 GC‐biased gene conversion links the recombination landscape and demography to genomic base composition: GC‐biased gene conversion drives genomic base composition across a wide range of species. BioEssays News Rev. Mol. Cell. Dev. Biol. 37:1317–1326. 10.1002/bies.201500058 26445215

[evl346-bib-0047] Nachman, M. W. , and B. A. Payseur . 2012 Recombination rate variation and speciation: theoretical predictions and empirical results from rabbits and mice. Philos. Trans. R. Soc. B Biol. Sci. 367:409–421. 10.1098/rstb.2011.0249 PMC3233716 22201170

[evl346-bib-0048] Nadeau, N. J. , S. H. Martin , K. M. Kozak , C. Salazar , K. K. Dasmahapatra , J. W. Davey , et al. 2013 Genome‐wide patterns of divergence and gene flow across a butterfly radiation. Mol. Ecol. 22:814–826. 22924870 10.1111/j.1365-294X.2012.05730.x

[evl346-bib-0049] Noor, M. A. , and S. M. Bennett . 2009 Islands of speciation or mirages in the desert? Examining the role of restricted recombination in maintaining species. Heredity 103:439–444. 19920849 10.1038/hdy.2009.151 PMC2809014

[evl346-bib-0050] Nosil, P. , and J. L. Feder . 2012 Genomic divergence during speciation: causes and consequences. Philos. Trans. R. Soc. B Biol. Sci. 367:332–342. 10.1098/rstb.2011.0263 PMC3233720 22201163

[evl346-bib-0051] Nosil, P. , D. J. Funk , and D. Ortiz‐Barrientos . 2009 Divergent selection and heterogeneous genomic divergence. Mol. Ecol. 18:375–402. 19143936 10.1111/j.1365-294X.2008.03946.x

[evl346-bib-0052] Paradis, E. , S. R. Baillie , W. J. Sutherland , and R. D. Gregory . 1998 Patterns of natal and breeding dispersal in birds. J. Anim. Ecol. 67:518–536.

[evl346-bib-0053] Pereira, R. J. , F. S. Barreto , N. T. Pierce , M. Carneiro , and R. S. Burton . 2016 Transcriptome‐wide patterns of divergence during allopatric evolution. Mol. Ecol. 25:1478–1493. 26859844 10.1111/mec.13579

[evl346-bib-0054] Phung, T. N. , C. D. Huber , and K. E. Lohmueller . 2016 Determining the effect of natural selection on linked neutral divergence across species. PLoS Genet. 12:e1006199. 27508305 10.1371/journal.pgen.1006199 PMC4980041

[evl346-bib-0055] Price, T. 2008 Speciation in birds. Roberts and Company Publishers, Colorado.

[evl346-bib-0056] Ravinet, M. , A. Westram , K. Johannesson , R. Butlin , C. André , and M. Panova . 2016 Shared and nonshared genomic divergence in parallel ecotypes of *Littorina saxatilis* at a local scale. Mol. Ecol. 25:287–305. 26222268 10.1111/mec.13332

[evl346-bib-0057] Renaut, S. , C. J. Grassa , S. Yeaman , B. T. Moyers , Z. Lai , N. C. Kane , et al. 2013 Genomic islands of divergence are not affected by geography of speciation in sunflowers. Nat. Commun. 4:1827. 23652015 10.1038/ncomms2833

[evl346-bib-0058] Renaut, S. , G. L. Owens , and L. H. Rieseberg . 2014 Shared selective pressure and local genomic landscape lead to repeatable patterns of genomic divergence in sunflowers. Mol. Ecol. 23:311–324. 26010734 10.1111/mec.12600

[evl346-bib-0059] Rosenzweig, B. K. , J. B. Pease , N. J. Besansky , and M. W. Hahn . 2016 Powerful methods for detecting introgressed regions from population genomic data. Mol. Ecol. 25:2387–2397. 26945783 10.1111/mec.13610 PMC4899106

[evl346-bib-0060] Singhal, S. , E. M. Leffler , K. Sannareddy , I. Turner , O. Venn , D. M. Hooper , et al. 2015 Stable recombination hotspots in birds. Science 350:928–932. 26586757 10.1126/science.aad0843 PMC4864528

[evl346-bib-0061] Smeds, L. , C. F. Mugal , A. Qvarnström , and H. Ellegren . 2016 High‐resolution mapping of crossover and non‐crossover recombination events by whole‐genome re‐sequencing of an avian pedigree. PLOS Genet. 12:e1006044. 27219623 10.1371/journal.pgen.1006044 PMC4878770

[evl346-bib-0062] Smukowski, C. S. , and M. A. F. Noor . 2011 Recombination rate variation in closely related species. Heredity 107:496. 21673743 10.1038/hdy.2011.44 PMC3242630

[evl346-bib-0063] Stapley, J. , T. R. Birkhead , T. Burke , and J. Slate . 2008 A linkage map of the zebra finch *Taeniopygia guttata* provides new insights into avian genome evolution. Genetics 179:651–667. 18493078 10.1534/genetics.107.086264 PMC2390641

[evl346-bib-0064] Toews, D. P. , S. A. Taylor , R. Vallender , A. Brelsford , B. G. Butcher , P. W. Messer , et al. 2016 Plumage genes and little else distinguish the genomes of hybridizing warblers. Curr. Biol. 26:2313–2318. 27546575 10.1016/j.cub.2016.06.034

[evl346-bib-0065] Turner, T. L. , and M. W. Hahn . 2010 Genomic islands of speciation or genomic islands and speciation? Mol. Ecol. 19:848–850. 20456221 10.1111/j.1365-294X.2010.04532.x

[evl346-bib-0066] Vallender, R. , R. J. Robertson , V. L. Friesen , and I. J. Lovette . 2007 Complex hybridization dynamics between golden‐winged and blue‐winged warblers (*Vermivora chrysoptera* and *Vermivora pinus*) revealed by AFLP, microsatellite, intron and mtDNA markers. Mol. Ecol. 16:2017–2029. 17498229 10.1111/j.1365-294X.2007.03282.x

[evl346-bib-0067] Van Doren, B. A. , L. Campagna , B. Helm , J. Illera , I. J. Lovette , and M. Liedvogel . 2017 Correlated patterns of genetic diversity and differentiation across an avian family. Mol. Ecol. 26:3982–3997. 28256062 10.1111/mec.14083

[evl346-bib-0068] Vijay, N. , M. Weissensteiner , R. Burri , T. Kawakami , H. Ellegren , and J. B. Wolf . 2017 Genome‐wide patterns of variation in genetic diversity are shared among populations, species and higher order taxa. Mol. Ecol. 10.1111/mec.14195 28570015

[evl346-bib-0069] Warren, W. C. , D. F. Clayton , H. Ellegren , A. P. Arnold , L. W. Hillier , A. Künstner , et al. 2010 The genome of a songbird. Nature 464:757–762. 20360741 10.1038/nature08819 PMC3187626

[evl346-bib-0070] Wu, C.‐I. 2001 The genic view of the process of speciation. J. Evol. Biol. 14:851–865.

[evl346-bib-0071] Zhang, G. , E. D. Jarvis , and M. T. P. Gilbert . 2014 A flock of genomes. Science 346:1308–1309. 25504710 10.1126/science.346.6215.1308 PMC4407557

