# Supplementary material for: Comparative analysis examining patterns of genomic differentiation across multiple episodes of population divergence in birds
Source: Evol Lett. 2018 Mar 15;2(2):76–87. doi: 10.1002/evl3.46 (PMC6121856; doi:10.1002/evl3.46)
Supplement: Supplementary file 1 — Table S1. Datasets included in the present study. Table S2. Coverage of consensus genomes. Table S3. Repeatability measured using outlier status and overlap values. Table S4. Data used to measure the speciation continuum. [file EVL3-2-76-s001.docx]

**Table of Contents**

Table S1. Datasets included in the present study.

Table S2. Coverage of consensus genomes.

Table S3. Repeatability measured using outlier status and overlap values.

Table S4. Data used to measure the speciation continuum.

Table S5. Results from GLMs examining the relationship between repeatability and predictor variables related to genomic features.

Supplementary Methods.

Table S1. Datasets included in the present study.

| Species | *Ficedula* flycatchers | *Corvus* crows | *Phylloscopus* greenish warblers | *Catharus* thrushes | *Phylloscopus* willow warblers | *Saxicola* stonechats | *Sylvia* blackcaps | *Vermivora* warblers |
| --- | --- | --- | --- | --- | --- | --- | --- | --- |
| Pair | Collared and pied (*F. albicollis* and *hypoleuca*) | Hooded and carrion (*C. corone orone* and *C. c. cornix*) | Eastern and western (*P. tochiloides plumbeitarsus* and *P. t.* *viridanus*) | Coastal and inland (*Catharus ustulatus ustulatus* and *C. u. swainsoni*) | Southern and northers (*P. trochilus trochilus* and *P. t. acredula*) | European and Siberian (*S. rubicola* and *S. maurus*) | German and Austrian populations (*Sylvia atricapilla*) | Blue- and golden-winged (*V. cyantoptera* and *V. chrysoptera*) |
| Resequencing data | WGS data from four populations (n=20/population) | WGS data from two populations (n=15/population) | GBS data from 34 and 19 birds, respectively | WGS poolseq data from 2 populations (n=10/population) | WGS data from two populations (n=9/population) | WGS poolseq data (n=49 [European] and 52[Siberian]) | WGS data from two populations (n=15/population) |  |
| Accession numbers | ENA PRJEB7359; Genbank AGT002000000 | SRA PRJNA192205 | SRA SRR1176844; DDBJ/ENA/GenBank LYPA00000000 | SRA PRJNA275819 | SRP074112 | ENA PRJEB19452; PRJEB19453 | https://www.zoology.  ubc.ca/~kdelmore/; Dryad upon acceptance. | PRJNA325126; PRJNA325157 |
| References | Burri et al. 2015  Kawakami et al. 2014 | Poelstra et al. 2014 | Alcaide et al. 2014; Irwin et al. 2016 | Delmore et al. 2015 | Lundberg et al. 2017 | Van Doren et al. 2017 | Delmore et al. in prep | Toews et al. 2016 |

Table S2. Summary of final consensus sequences included in the present study. The length of each chromosome is provided along with the percentage of the flycatcher chromosome it covers. Lengths are the number of base pairs in the consensus without gaps of Ns. Macrochromosomes are those greater than 40 Mb and microchromosomes those less than 20 Mb.

| chr | **flycatcher** | blackcap | | crow | | blue/gold | | willow | | thrush | | greenish | | stonechat | |
| --- | --- | --- | --- | --- | --- | --- | --- | --- | --- | --- | --- | --- | --- | --- | --- |
|  | **length** | length | % | length | % | length | % | Length | % | length | % | length | % | length | % |
| 1 | **120002344** | 90136271 | 75.1 | 59620835 | 49.7 | 95993241 | 80.0 | 54058012 | 45.0 | 97347748 | 81.1 | 97683349 | 81.4 | 102066987 | 85.1 |
| 1A | **74947036** | 48879787 | 65.2 | 40202034 | 53.6 | 57498691 | 76.7 | 57749718 | 77.1 | 58550819 | 78.1 | 61028700 | 81.4 | 63356200 | 84.5 |
| 2 | **157563209** | 132248109 | 83.9 | 73562279 | 46.7 | 122799733 | 77.9 | 116957659 | 74.2 | 129397975 | 82.1 | 130746161 | 83.0 | 117858622 | 74.8 |
| 3 | **115844353** | 96698794 | 83.5 | 75853557 | 65.5 | 90728830 | 78.3 | 92584803 | 79.9 | 96761318 | 83.5 | 97746071 | 84.4 | 97985779 | 84.6 |
| 4 | **70439523** | 52016224 | 73.8 | 60250282 | 85.5 | 56882071 | 80.8 | 37147958 | 52.7 | 58149848 | 82.6 | 59354464 | 84.3 | 62340083 | 88.5 |
| 4A | **21182716** | 16026903 | 75.7 | 17022485 | 80.4 | 15762793 | 74.4 | 15154164 | 71.5 | 15488001 | 73.1 | 14334247 | 67.7 | 18296289 | 86.4 |
| 5 | **64724594** | 49907695 | 77.1 | 52762582 | 81.5 | 48862770 | 75.5 | 54367822 | 84.0 | 51849320 | 80.1 | 53034140 | 81.9 | 56805964 | 87.8 |
| 6 | **37227452** | 31384756 | 84.3 | 25164402 | 67.6 | 30082679 | 80.8 | 29498648 | 79.2 | 30044214 | 80.7 | 30396279 | 81.7 | 33408554 | 89.7 |
| 7 | **39412007** | 34094738 | 86.5 | 33361110 | 84.6 | 31303551 | 79.4 | 34263494 | 86.9 | 32013904 | 81.2 | 33078282 | 83.9 | 34858256 | 88.4 |
| 8 | **32100816** | 22935871 | 71.4 | 27830922 | 86.7 | 26078388 | 81.2 | 28020258 | 87.3 | 25395769 | 79.1 | 26648552 | 83.0 | 28513982 | 88.8 |
| 9 | **26793321** | 22576347 | 84.3 | 22910380 | 85.5 | 21086208 | 78.7 | 21779656 | 81.3 | 20931760 | 78.1 | 21800432 | 81.4 | 21898138 | 81.7 |
| 10 | **21346708** | 17924858 | 84.0 | 16798936 | 78.7 | 16978521 | 79.5 | 17865715 | 83.7 | 16663500 | 78.1 | 17498509 | 82.0 | 18776919 | 88.0 |
| 11 | **21727166** | 18258423 | 84.0 | 12507783 | 57.6 | 17216928 | 79.2 | 18746169 | 86.3 | 16691697 | 76.8 | 17523717 | 80.7 | 19428494 | 89.4 |
| 12 | **21938106** | 18236025 | 83.1 | 18666245 | 85.1 | 17207319 | 78.4 | 18665559 | 85.1 | 17090405 | 77.9 | 17265384 | 78.7 | 19429616 | 88.6 |
| 13 | **18641552** | 15235186 | 81.7 | 14485192 | 77.7 | 14290123 | 76.7 | 14820107 | 79.5 | 13540864 | 72.6 | 14345885 | 77.0 | 16149134 | 86.6 |
| 14 | **17374186** | 14026179 | 80.7 | 14416366 | 83.0 | 13296177 | 76.5 | 13987910 | 80.5 | 12888791 | 74.2 | 13233457 | 76.2 | 15145780 | 87.2 |
| 15 | **14943019** | 12117646 | 81.1 | 9640549 | 64.5 | 11301992 | 75.6 | 12105690 | 81.0 | 10698802 | 71.6 | 10643530 | 71.2 | 12836525 | 85.9 |
| 17 | **12378331** | 9789432 | 79.1 | 9758513 | 78.8 | 7512843 | 60.7 | 9969948 | 80.5 | 8307378 | 67.1 | 8957804 | 72.4 | 10137087 | 81.9 |
| 18 | **13163162** | 9605582 | 73.0 | 7496181 | 56.9 | 8062768 | 61.3 | 9676338 | 73.5 | 8554042 | 65.0 | 8166354 | 62.0 | 10117730 | 76.9 |
| 19 | **11933672** | 9561990 | 80.1 | 9593105 | 80.4 | 8425778 | 70.6 | 9775189 | 81.9 | 8406962 | 70.4 | 8779264 | 73.6 | 10234172 | 85.8 |
| 20 | **15675940** | 12527461 | 79.9 | 12566710 | 80.2 | 11973761 | 76.4 | 12967530 | 82.7 | 11239349 | 71.7 | 12053729 | 76.9 | 12804409 | 81.7 |
| 21 | **8073070** | 5742695 | 71.1 | 5944624 | 73.6 | 4230261 | 52.4 | 5818547 | 72.1 | 4709579 | 58.3 | 5100060 | 63.2 | 6288065 | 77.9 |
| 22 | **5733621** | 2396835 | 41.8 | 3162411 | 55.2 | 1871261 | 32.6 | 2718128 | 47.4 | 2053051 | 35.8 | 1540315 | 26.9 | 3592435 | 62.7 |
| 23 | **7944683** | 5308793 | 66.8 | 5126807 | 64.5 | 4132107 | 52.0 | 4646607 | 58.5 | 3739029 | 47.1 | 3522204 | 44.3 | 5559624 | 70.0 |
| 24 | **8009359** | 5796045 | 72.4 | 5988272 | 74.8 | 5037427 | 62.9 | 5997533 | 74.9 | 4496957 | 56.1 | 4707706 | 58.8 | 6516443 | 81.4 |
| 25 | **2802420** | 1025451 | 36.6 | 963319 | 34.4 | 721357 | 25.7 | 978166 | 34.9 | 682990 | 24.4 | 369145 | 13.2 | 1215130 | 43.4 |
| 26 | **7653694** | 4938326 | 64.5 | 4968167 | 64.9 | 3648646 | 47.7 | 4565093 | 59.6 | 3510613 | 45.9 | 3577204 | 46.7 | 3938873 | 51.5 |
| 27 | **5572044** | 3195560 | 57.3 | 3092758 | 55.5 | 2563901 | 46.0 | 3153518 | 56.6 | 2546310 | 45.7 | 2545477 | 45.7 | 3727994 | 66.9 |
| 28 | **6182350** | 3690716 | 59.7 | 3728152 | 60.3 | 2782648 | 45.0 | 3995888 | 64.6 | 2898680 | 46.9 | 2931216 | 47.4 | 4436153 | 71.8 |
| Z | **59856998** | 45300926 | 75.7 | 37259879 | 62.2 | 46372801 | 77.5 | 45029809 | 75.2 | 47742596 | 79.8 | 48790136 | 81.5 | 48563346 | 81.1 |
| **Total** | **1041187452** | **811583624** | **77.9** | **684704837** | **65.8** | **794705574** | **76.3** | **757065636** | **72.7** | **812392271** | **78.0** | **827401773** | **79.5** | **866286783** | **83.2** |

**Table S3.** Summarizing repeatability in genomic differentiation across pairs using outlier status of windows and observed values of overlap. Values in (a) are z-scores, comparing the number of outlier windows that were shared across each comparison to the mean expected number obtained using the hypergeometric distribution. Z-scores are effect sizes that indicate how many standard deviations the observed value is beyond the mean expected value. Values in (b) are based on the same outlier windows, but combining strings of outliers into peaks and expressing estimates of overlap as the total number of shared peaks in the comparison divided by the total number of unique peaks in the comparison. Results for *F_ST_* are shown below the diagonal and *d_XY_* above (not estimated for greenish warbelrs).

a)

|  | Flycatcher | Crows | Willows | Blackcaps | Greenish | Stonechats | Thrushes | Blue/gold |
| --- | --- | --- | --- | --- | --- | --- | --- | --- |
| Flycatcher |  | 0.92 | 0.40 | 1.23 |  | 3.56*** | 1.30 | 0.62 |
| Crows | 0.22 |  | 0.25 | 1.60* |  | 1.60* | 1.60* | 0.55 |
| Willows | -0.44 | -0.39 |  | 2.35* |  | 0.77 | 1.98* | 2.20** |
| Blackcaps | -0.22 | 0.06 | 0.72 |  |  | 2.96*** | 4.24*** | 4.01*** |
| Greenish | 2.16* | 0.39 | -0.06 | 0.22 |  |  |  |  |
| Stonechats | 1.88* | 0.61 | 0.17 | -0.11 | 1.22 |  | 4.76*** | 1.07 |
| Thrushes | 1.00 | -0.17 | 1.33 | 0.39 | 0.78 | 1.50 |  | 2.58*** |
| Blue/gold | -0.55 | 0.17 | 1.33 | 0.66 | 0.55 | -0.66 | 1.66* |  |

b)

|  | Flycatcher | Crows | Willows | Blackcaps | Greenish | Stonechats | Thrushes | Blue/gold |
| --- | --- | --- | --- | --- | --- | --- | --- | --- |
| Flycatcher |  | 0.08* | 0.15** | 0.14* |  | 0.2*** | 0.16*** | 0.18*** |
| Crows | 0.04 |  | 0.09*** | 0.10** |  | 0.06* | 0.06* | 0.08** |
| Willows | 0.04 | 0.04 |  | 0.22*** |  | 0.10*** | 0.20* | 0.20* |
| Blackcaps | 0.04 | 0.06 | 0.07 |  |  | 0.14*** | 0.22*** | 0.32** |
| Greenish | 0.09* | 0.05 | 0.09 | 0.07 |  |  |  |  |
| Stonechats | 0.15*** | 0.08 | 0.04 | 0.05 | 0.11** |  | 0.10*** | 0.12* |
| Thrushes | 0.05 | 0.05 | 0.07 | 0.06 | 0.08 | 0.08 |  | 0.23*** |
| Blue/gold | 0.03 | 0.06 | 0.1 | 0.09 | 0.06 | 0.04 | 0.08* |  |

p-values corrected for multiple testing (* 0.05, ** 0.01, *** 0.001)

Table S4. Variables used to estimate the speciation continuum, including genetic distance based on cytb and autosomal sequences, hybrid zone width and the proportion of hybrids in each zone (the latter variable is missing for European blackcaps).

|  | Cytb | Autosomal | Width (km) | Proportion of hybrids |
| --- | --- | --- | --- | --- |
| Flycatchers | 0.036 | 0.0035 | 20 ^a^ | 3^a^ |
| Crows | 0.029 | 0.00044 | 67^b^ | 12 ^a^ |
| Willows | 0.003 | 0.0017 | 350 ^a^ | 70^g^ |
| Blackcaps | 0.027 | 0.0021 | 340^c^ |  |
| Greenish | 0.051 | 0.0032 | 0^d^ | 0 ^d^ |
| Stonechats | 0.035 | 0.0039 | 0^e^ | 0^e^ |
| Thrushes | 0.048 | 0.0044 | 50^f^ | 20.5 ^f^ |
| Blue/golden-winged | 0.027 | 0.0029 | 600 ^a^ | 0^h^ |

^a^ Price 2008

^b^ Haas And Brodin 2005

^c^ Berthold et al. 1990

^d^ Alcaide et al. 2014

^e^ Helm 2009

^f^ Ruegg 2009

^g^ Lundberg et al. 2017

^h^ Vallender et al. 2007

**Supplementary Methods** – Additional details on how *d_s_* was estimated.

We performed gene prediction for each consensus genome with the MAKER pipeline which included four rounds of gene prediction as follows. The first round included gene prediction with EXONERATE using cDNA transcripts retrieved from Ensembl for zebra finch, chicken and flycatchers. This round also included repeat masking using the library of “aves” included in REPEATMASKER. For the second round, an HMM model was obtained from all gene predictions to use as input for the gene predictor SNAP. An additional round of repeat masking was run as described before. Third and fourth rounds of MAKER included two gene predictors: SNAP using HMM models from the previous round and the “chicken” HMM model available in AUGUSTUS. In every iteration we accepted only models with start and finish codons and genes > 50 amino acid (AA) length. Once we had annotated each consensus genome, we identified potential homologues for high quality transcripts (AED < 0.05) using a Blastn search against all transcripts from the flycatcher (flycatcher was searched against zebra finch). In this search we obtained the best hit of a transcript with at least 60% of identity and coverage of at least 50% of the flycatcher transcript. We then aligned codons from each pair of sequences using PRANK to calculate *d_N_*/*d_s_* with PAML v4.8 package. All *d_N_*/*d_s_* calculations were performed pairwise, comparing all the species with the flycatcher and this in turn, compared to zebra finch. We extracted only *d_s_* values from this analysis and to avoid false positives and (potential mistakes in alignments) we filtered out results with *d_s_* values bigger than 2 SD.
